# Supplementary material for: Quantifying the role of surface plasmon excitation and hot carrier transport in plasmonic devices
Source: Nat Commun. 2018 Aug 23;9:3394. doi: 10.1038/s41467-018-05968-x (PMC6107582; doi:10.1038/s41467-018-05968-x)
Supplement: Supplementary file 1 — Supplementary Information [file 41467_2018_5968_MOESM1_ESM.pdf]

Supplementary Information for:

Quantifying the Role of Surface Plasmon Excitation and Hot Carrier Transport in Plasmonic Devices

Giulia Tagliabue et al.

Supplementary Note 1: Polarization Dependence

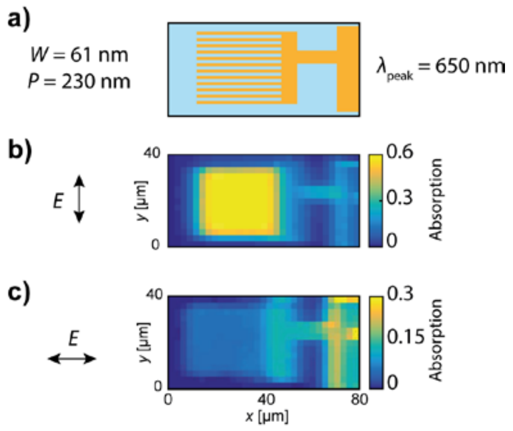

Supplementary Figure 1: Polarization Dependence. a) Schematic of the structure; b,c) absorption spatial maps at the resonance wavelength, λ_{peak} = 650 nm, for polarization perpendicular (b) and parallel (c) to the stripes. The latter two, clearly show the plasmonic nature of the mode, which enhances absorption only for perpendicular polarization.

Supplementary Note S2: Optical (R,T,A) and Photoelectrical (Responsivity, EQE) Characterization

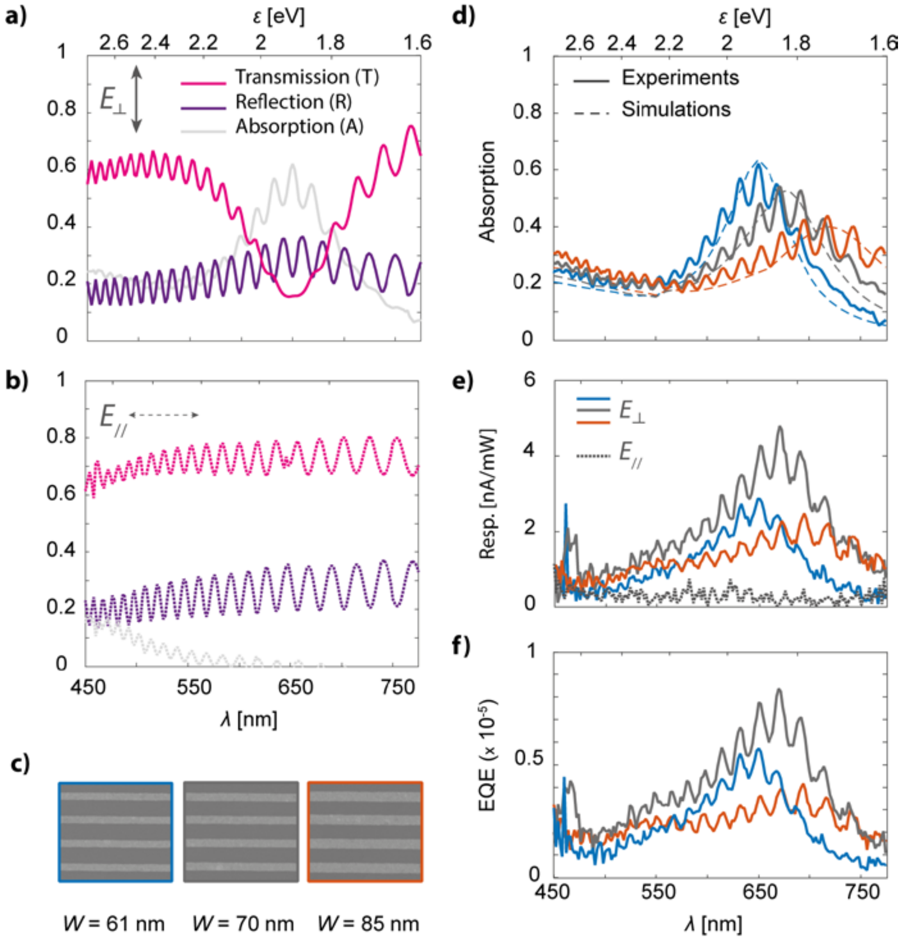

Supplementary Figure 2 : Optical and Photoelectrical Characterization. Transmission (T) and reflection (R) spectra of the device with stripe width W = 61 nm measured with a) light polarized perpendicular to the stripes and b) parallel to the stripes. The light grey curves show the experimental absorption spectrum determined as A = 1 – T - R; c) SEM micrographs of the three fabricated devices; d) Experimental absorption spectra of the three fabricated devices. Colors correspond to part c). The dashed lines show the corresponding absorption spectra calculated from COMSOL simulations; e) Responsivity spectra of the three plasmon-enhanced IPE photo-electrodes. The dashed curve shows the responsivity upon polarization parallel to the stripes; f) External quantum efficiency spectra (EQE) of the three IPE photo-electrodes;

Supplementary Note 3: Interplay of the metal band structure and interface properties on IQE

In Figure 3 of the manuscript, we reported the energy distributions of hot-electrons generated in Au for different photon energies (Figure 3a,b), as obtained from ab-initio calculations, and we discussed how the interplay with a Schottky barrier of ~1.2 eV determines the salient features of the experimentally observed IQE spectra (Figure 3c). Here we extend our analysis to different values of the Schottky barrier.

Supplementary Figure 3a shows the calculated IQE for an Au/semiconductor interface (electron effective mass of GaN ~0.27 m_{0e}[1] as a function of the interfacial Schottky barrier height. Beyond the specificity of each curve we observe that some features are common to all the spectra:

- At low photon energies (close to the Schottky barrier) the IQE grows rapidly, roughly following the expected free electron-like Fowler behavior
- Around 1.7eV, the IQE growth is suppressed and the curve deviates significantly from the Fowler model
- Beyond ~2.3-2.4 eV the IQE exhibits a peak and then a pronounced drop

These spectral features of the IQE are directly associated with the Au electronic band structure:

- Up to ~1.6 eV, intraband transitions dominate hot carrier excitation. For this reason the IQE spectrum follows closely the Fowler model, which accounts only for free-electron energy distributions;
- Between 1.6 eV and 1.8 eV, interband transitions start to contribute significantly and the IQE deviates more prominently from the free-electron like Fowler model. The suppression of IQE can be understood by looking at Supplementary Figure 3. Indeed, starting at around 1.7 eV, interband transitions induce a redistribution of the energy of the hot-electrons towards lower energies, close to the metal Fermi level. This reduces the number of high-energy electrons that can be collected with better efficiency than low-energy electrons and suppresses the IQE compared to expectations based solely on the Fowler model.
- Around ~2.3-2.4 eV, interband transitions finally become the dominant mechanism for hot-electrons generation leading to a sharper drop in IQE, which then reaches a minimum around 2.6 eV.

The effect of the interplay between intra and interband transitions can be entirely explained by considering the metal electronic band structure. On the other hand, the energy filtering effect of the metal/semiconductor interface, which combines the energetics of the Schottky barrier and momentum matching conditions, modulates the relative prominence of these three different regimes (intraband regime up to 1.6 eV, mixed regime 1.7-2.3 eV, interband regime above 2.4 eV). In fact, we observe that:

- For a Schottky barrier of 0.9 eV, effective collection of high-energy electrons from intraband transitions leads to a peak in IQE around 1.7 eV, right before the onset of interband transitions. A slow decrease in IQE is observed in the mixed regime before a sharp drop-off occurs around 2.4 eV;
- For a Schottky barrier of 1.2 eV, the collection of hot electrons in the intraband regime is reduced and therefore the mixed regime between 1.7 eV and 2.3 eV still exhibits a slow growth of IQE. Indeed, the increased injection probability of the highest energy electrons compensates the overall reduced probability of intraband generated hot-electrons. Only the complete transition to the interband regime around 2.4 eV suppresses the IQE;
- For an even larger Schottky barrier of 1.5 eV, carrier collection is negligible across the entire intraband regime. Therefore the transition between the Fowler-like rapid growth in IQE and the IQE reduction due to interband transitions cannot be observed. Instead, throughout the mixed regime, IQE slowly grows thanks to the highly energetic fraction of hot-electrons generated through intraband transitions. Transition into the interband regime around 2.4 eV, however, halts this growth.

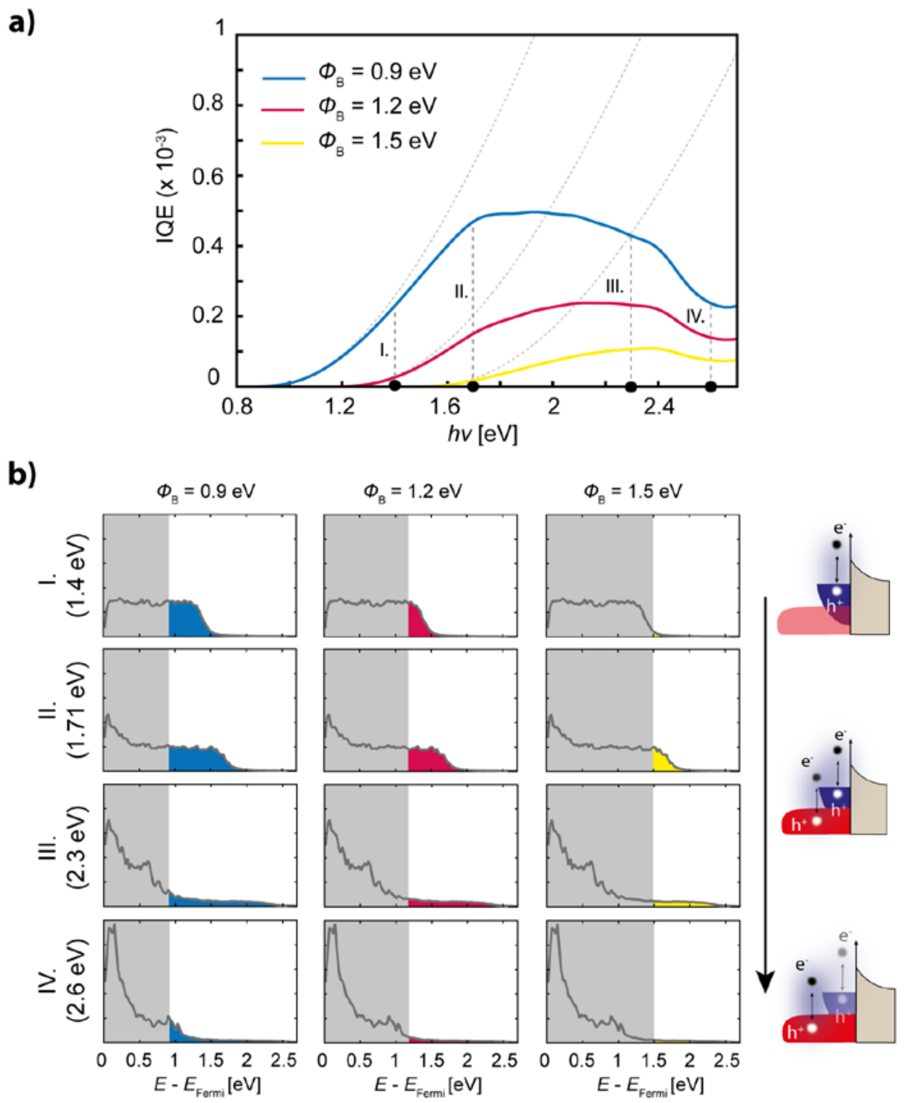

Supplementary Figure 3: Interplay of metal band structure and Schottky barrier in the IQE. a) IQE of an Au/semiconductor (electron effective mass of GaN ~0.27 m_{0e}) device as a function of the Schottky barrier height. The light-grey dashed lines represent the IQE predicted by the Fowler model. The dark-grey vertical dashed lines identify the four photon energies analyzed in part b; b) hot carrier energy distribution for four different photon energies (from top to bottom: 1.4 eV, 1.71 eV, 2.3 eV and 2.6 eV). Each column corresponds to a different Schottky barrier height (from left to right: 0.9 eV, 1.2 eV, 1.5 eV) and shows how the energetics of the interface filter the hot carrier energy distribution.

From this analysis we observe that due to the combined effect of the metal band structure and the interface, the IQE spectrum can exhibit a peak in the photon energy range anywhere between ~ 1.8 eV to 2.4 eV. Although small gold nanoparticles or thin Au films can exhibit a plasmon resonance in this very same energy range, these two aspects should not be confounded. Indeed, plasmon excitation will affect the optical properties of the system and manifest itself in an enhanced external quantum efficiency of the device. However the peak in IQE originates solely from the electronic properties of the metal, with modulations induced by the characteristics of the metal/semiconductor interface.

Supplementary Note 4: Detailed analysis of IQE - Injection Probability

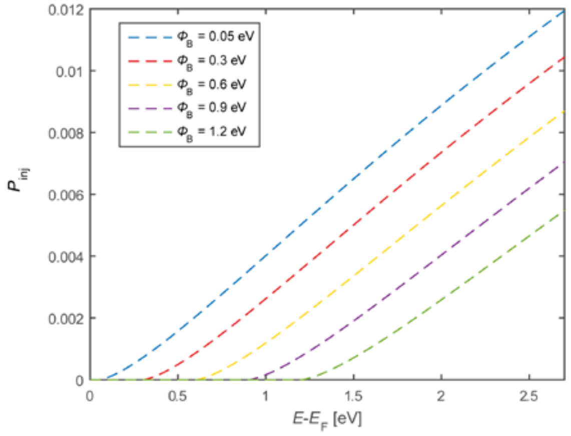

Supplementary Figure 4: Injection probability. Calculated injection probability including momentum matching and tangential momentum conservation across the metal-semiconductor interface shown for an increasing value of the barrier height, Φ_B. The low values originate from the low effective electron mass (~$0.27m_{0e}$) in the close-to-ideal, parabolic conduction band of GaN and from the smooth metal-semiconductor interface imposing tangential momentum conservation on hot electron injection.

Supplementary Note 5: Detailed analysis of IQE - Transport vs Barrier Height

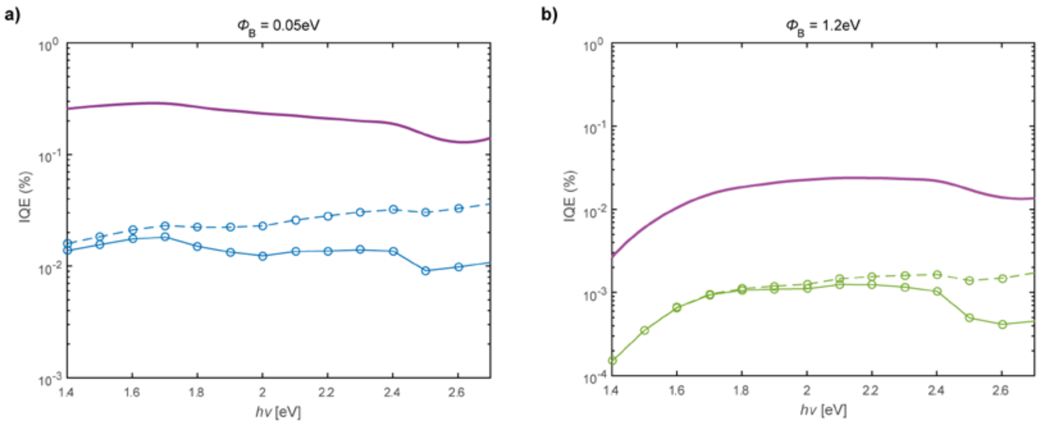

Supplementary Figure 5: IQE as a function of Schottky barrier height and transport for Au/n-GaN interfaces. a) Φ_B = 0.05 eV : purple curve - expected IQE accounting for momentum matching (P_{inj}) but not for transport; blue curve IQE accounting for transport (solid - ballistic injection; dashed – N=3); b) Φ_B = 1.2 eV : purple curve - expected IQE accounting for momentum matching but not for transport; green curve IQE accounting for transport (solid - ballistic injection; dashed – N=3);

Supplementary Figure 5a shows that even with a negligible Schottky barrier of 50 meV, momentum-matching conditions set a very tight restriction on IQE, limiting the efficiency to less than 1% across the entire visible spectrum (purple curve). Transport effects, meaning scattering and recombination of carriers in the structure with finite dimensions, further reduce IQE by one to two orders of magnitude (blue solid line). In particular, in the case of Au, IQE decreases with the onset of interband transitions (~1.8eV) due to the lower probability of injection across the Au/GaN interface for carriers with smaller energies (Figure 3 and Supplementary Figure 4). Collection of scattered carriers offers only a marginal improvement of IQE (blue dashed line) for the same reason. A larger barrier (Supplementary Figure 5b) exacerbates these mechanisms. Therefore improving the injection efficiency across the interface and ensuring ballistic collection are of primary importance, irrespective of the barrier height of a given metal-semiconductor junction. In this respect, roughening of the interface and choice of semiconductors with high density of states in the conduction band (e.g. TiO_2) would be highly beneficial.

Supplementary Note 6: I-t trace of short circuit current and dark I-V response

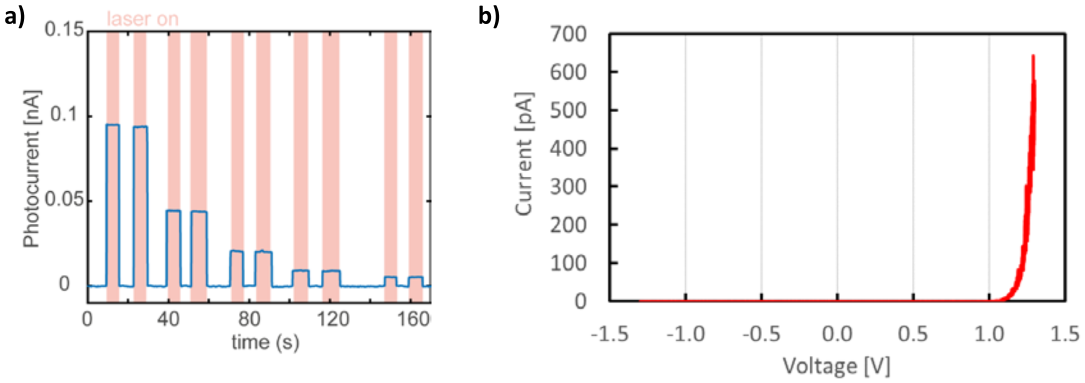

Supplementary Figure 6: Photocurrent and I-V characteristics a) Photocurrent-time (*I*-*t*) trace of the plasmonic hot carrier device showing the short circuit current upon illumination with a 633nm laser at different powers (same as Figure 2b); b) *I-V* response of the device showing the highly rectifying behavior associated with the presence of a Schottky barrier of approximately 1.2eV.

Supplementary Note 7: Fabry-Perot modelling

In a Fabry-Perot etalon the peaks in transmission correspond to dips in reflection and vice versa. The peaks occur when the phase accumulation of the light wave during a round-trip across the high-index region (GaN in our case) is equal to 2π (constructive interference). If the refractive index and thickness of the GaN layer are n and t, respectively, we can calculate the phase accumulation as: $\delta = \frac{2\pi}{\lambda} n \cdot 2t\cos\theta$ where θ is the angle of incidence of the incident light beam. Therefore, the peak wavelengths can be identified by the condition: $\frac{\delta}{2\pi} = m = \frac{n(\lambda)\cdot 2t\cos\theta}{\lambda_{peak}}$ where *m* must be an integer number and we have also included the wavelength-dependence of the refractive index, *n(λ)*.

From our measurements we actually have a series of $\lambda_{\mathrm{peak,exp}}$ values, and can therefore find a thickness *t* that correctly predicts our experimental peak series. As can be observed in the Supplementary Figure 7.a, when *t* = 4228 nm ≈ 4.23 µm we can match very well the calculated Fabry-Perot transmission peaks (vertical dashed lines) with the peaks in the experimental transmission spectrum (red curve) or the dips in the experimental reflection spectrum (blue curve). According to the manufacturer, our GaN substrates have a nominal thickness of t_{nominal} = 4 ± 1 µm and therefore the estimated thickness *t* is in excellent agreement with the expected one. Small deviations can be attributed to i) minor variations between the used values of *n(λ)*, that we took from the literature, and the refractive index of our specific substrate and ii) the finite resolution of our experimental spectra (acquired with 2 nm resolution).

In order to reduce computation time as well as improve clarity of data display, in Figure 2e and Supplementary Figure 2c we have reported the absorption calculated using a thin GaN substrate (500 nm thick), which does not display Fabry-Perot oscillations. In Supplementary Figure 7.b we show the result of a numerical simulation where we have included the full GaN substrate: as expected we observe Fabry-Perot oscillations in the absorption spectrum.

Lastly we note that Fabry-Perot interference is extremely sensitive to the light path within the high refractive index material (in our case GaN). The incomplete cancellation of the Fabry-Perot fringes in the IQE spectra (Figure 2g) is attributable to differences in the angular alignment of ±3 degrees between the IQE and absorption measurements, which were done in separate experimental setups. The plasmonic absorption is instead insensitive to angular

variations of this magnitude. Therefore, the small discrepancy in Fabry-Perot fringes, which leads to their incomplete cancellation, has no bearing on the overall IQE measurement.

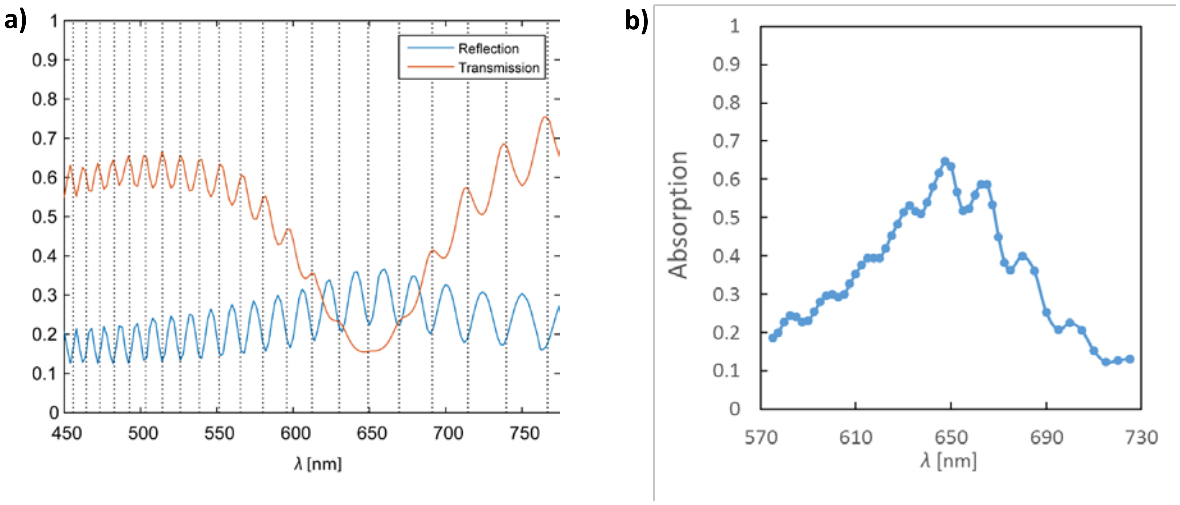

Supplementary Figure 7: **Fabry-Perot Modelling**. a) Calculated Fabry-Perot resonance peaks (dashed black lines) and comparison to the experimental transmission (red) and reflection (blue) curves; b) calculated absorption (COMSOL) for a geometry where the full GaN layer has been included.

Supplementary Note 8: Field Enhancement

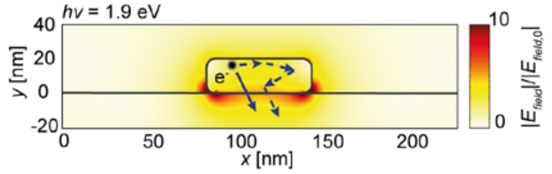

Supplementary Figure 8: Calculated field enhancement corresponding to the reported electric field norm in Figure 4a.

Supplementary References

[1] Rheinlander, Neumann, Phys. Status Solidi (b) 64, 1974
